# Does Traditional Feeding of Outdoor Guard Dogs Provide a Food Resource for Wild Mammals and Birds?

**DOI:** 10.3390/ani11051198

**Published:** 2021-04-22

**Authors:** Róża Andrzejczak, Łukasz Dylewski, Leszek Jerzak, Branislav Peťko, Łukasz Myczko

**Affiliations:** 1 Department of Zoology, Poznań University of Life Sciences, Wojska Polskiego 71 C, 60-625 Poznań, Poland; roza.andrzejczak@wp.pl (R.A.); brano.petko@gmail.com (B.P.); 2 Institute of Biological Scienes, University of Zielona Góra, 65-516 Zielona Góra, Poland; l.jerzak@wnb.uz.zgora.pl; 3 Institute of Dendrology, Polish Academy of Sciences, Parkowa 5, 62-035 Kórnik, Poland; dylewski91@gmail.com; 4 University of Veterinary Medicine in Kosice, Komenského 68/73, 041 81 Košice, Slovakia

**Keywords:** traditional food sources, farmland, pets, villages

## Abstract

**Simple Summary:**

Access to food is crucial in the life of birds, and affects reproduction, survival and, consequently, population size. We investigated how traditional care of dogs affected rural birds and other animal populations. Using camera traps, it was found that the food fed to dogs was also taken by seven species of birds and at least three species of mammals. The most numerous species taking dog food was the house sparrow, *Passer domesticus*, which is declining in Europe. In the case of this species, females were more likely than males to use food provided for dogs, with a clear preference for food prepared in the human kitchen. We conclude that the food provided to domestic pets can be an important component of the diet of wild birds and mammals living close to humans.

**Abstract:**

Access to food is crucial in the life of birds and affects reproduction, survival and, consequently, population size. In the case of bird species inhabiting villages, poorer food conditions now exist, mainly because of changes in the lifestyle of rural residents and a reduction in the number of farm animals traditionally housed in backyards. Recent changes have also affected dog populations in villages, and the majority of them are no longer kept outside as guard dogs, but rather inside houses as pets. We investigated how traditional care of dogs impacted rural birds and other animal populations. The study was carried out at the end of winter and early spring in 29 farmsteads in western Poland. Using camera traps, it was found that the food fed to dogs was also taken by seven species of birds and at least three species of mammals. The most numerous species taking dog food was the house sparrow, *Passer domesticus*, which is declining in Europe. In the case of this species, females were more likely than males to use food given to dogs, with a clear preference for food prepared in the human kitchen. We conclude that the food provided to domestic pets can be an important component of the diet of wild birds and mammals living close to humans.

## 1. Introduction

Birds in farmland are declining across Europe, and one of the main reasons is decreasing food resources resulting from the intensification of agriculture [1,2,3]. However, in Central Europe, the population size of birds in farmland is generally higher due to less-intensive agriculture [3,4], but also from the presence of refugia for many species in agricultural holdings, both in the breeding season [5], and throughout the year [6]. To date, research has focused on the roles of plant food sources; weeds on the outskirts of the village, and the remains of grain provided for poultry have been stressed [7]. Rural backyards were identified as being particularly important at the end of winter [8], when the availability of natural food sources is very limited, primarily as a result of the consumption of the best food sources [7]. The end of winter and the beginning of spring are the periods when birds replenish their energy resources which are necessary to start reproduction [9]. Therefore, accessing food during this period should involve the use of various available food resources. For example, feeding birds in gardens and yards is very common in villages and benefits many bird species [10]. But wild birds not only use food sources provided intentionally by humans, but also those provided unintentionally such as leftover feed provided to farm animals and waste food [7,10]. One example of the latter is food provided by farmers and owners to farm guard dogs [11]. The pattern of feeding dogs in villages has changed a lot recently and instead of food from the owner’s kitchen, more are fed with commercial food [12,13]. Moreover, due to sociological changes in villages [5,14], dogs are more often now typical pets, living in houses, rather than traditional guard animals [11]. Pet food is changing to have a greater proportion of plant ingredients, with a potentially visible impact on the environment [15]. However, Okin [15] did not discuss the link between dog food and wild animals. Obviously, birds foraging in farmyards will also try to use food provided for dogs. Therefore, the main aim of the current study was to describe the number of wild species of birds and mammals accessing food provided for dogs. In order to estimate the extent of this phenomenon and identify which species of birds and mammals were involved, our study took place in the critical period at the end of winter and in early spring. To avoid problems with species identification and the potential impact on wild animals (e.g., their behaviour, especially vigilance etc.) we decided to conduct the study in a non-invasive way using camera traps which have become popular in the study of the use of space by animals [16,17]. Videos from camera traps allowed us also to investigate the behaviour of wild animals using the food of guard dogs. Due to limits of population size, we decided to conduct a fuller analysis of the house sparrow, *Passer domesticus*, which has declined throughout Europe, partly due to lack of food, [18,19,20,21]. Additionally, we analyzed the influence of dog behaviour on the probability of house sparrow foraging. We predicted that aggressive behaviour of dogs would be treated by birds as a danger signal and would potentially negatively influence the probability of a visit [22]. Moreover, we expected that the response of birds to different dog behaviour, i.e., reaction or lack of reaction by feeding birds, would also be influenced by the sex of the bird [23].

## 2. Material and Methods

### 2.1. Fieldwork

The study was conducted in rural areas in western Poland, mainly in the vicinity of Poznań (52°24′ N, 16°55′ E). In total, 29 locations were selected where guard dogs were kept outside the property, and where owners regularly provided them with food and water. To avoid pseudo-replication, study locations were spaced apart by at least 1 km. Field work was carried out at the end of winter and early spring, i.e., at a critical period of availability of natural food sources for many farmland birds [6,9], from mid-February to the beginning of April 2016. At each location we set a Bushnell Camera Trap (NatureView HD Live View model No 119740), 1.5 m from, and aimed at, the dog food and water bowls. At each location the camera was installed for 24 h. Videos were initiated by a motion sensor and consisted of 30 s of movie with 1 s intervals [24]. The following parameters were collected at all locations: date, geographical coordinates, type of food fed to animals (purchased, home-prepared). Records were made of the type of bowl used by the observed animal (food bowl, water bowl), the place from where food was taken (bowl, food scattered), place of consumption (on the spot, taken away), and the species of the observed animal. In the case of house sparrows we determined the sex of the bird. We noted the dog’s reaction towards the animal taking its food if he ran to the bowl. We considered the dog reacted if it showed aggression more than once to animals taking food.

### 2.2. Statistical Analysis

We used a generalized linear mixed model (GLMM) with a negative binomial distribution to test the number of house sparrow visits compared to the type of supplementary resources, hereafter food type (home prepared food, purchased and water), sex (male or female), dog reaction (no-reaction, reaction). Additionally, we added the following interactions: food type × sex, food type × dog reaction and sex × dog reaction. We included location as a random factor. We used a least squares mean contrast with the Tukey method to compare between significant factor levels. For type of food, chi square contingency tests were used to compare the total numbers of visits between female and male house sparrow. All analyses were carried out in the R 3.3.2 statistical environment [25] using lme4 [26], lsmeans [27] and ggplot2 [28] packages.

## 3. Results

Wild animals were observed accessing dog food in 21 (72.4%) of the 29 locations. In total, 1818 visits by wild species were noted and in 1755 (96.5%) of these either food or water was taken. The mean (±SE) number of visits per [non-zero] location was 83.6 ± 23.6. Dog food was taken by wild animals on 1630 visits and water on 125 visits.

During the study, we recorded the use of dog food by seven species of birds (see Table 1) and at least three species of mammals including Red Fox *Vulpes vulpes*, Beech Marten *Martes foina* and mainly unidentified rodents. Most rodent recordings were night time recordings of small rodents lacking in characteristic features, thus we were not able to determine these to species level. However, recorded rodents included adult brown rats *Rattus norvegicus*. In other recordings young brown rats were likely candidates, but we cannot exclude the possibility of another rodent species (most likely house mouse *Mus musculus* or wood mouse *Apodemus sylvaticus*), and thus we decided to treat all rodents as a single group.

**Figure 1:**
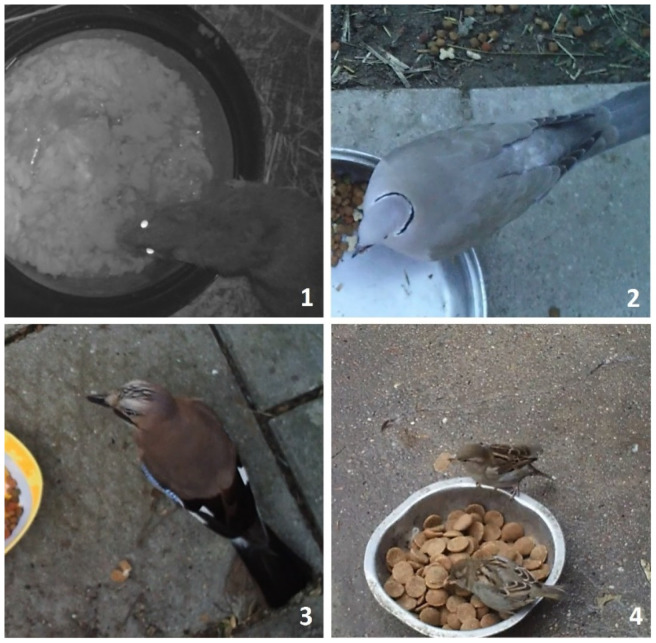
Examples of wild animals recorded with camera traps. (**1**) Brown rat *Rattus norvegicus*, (**2**) Collared dove *Streptopelia decaocto*, (**3**) Jay *Garrulus glandarius*, (**4**) female house sparrows *Passer domesticus*.

Recordings were dominated by house sparrow visits, comprising over 87% of all observed animals using dog food or water. For this reason, the more detailed analysis is focused only on this species. Out of 1584 recorded house sparrows visits, 898 (56.7%) were by females, and 686 (43.3%) by males (Table 2, Figure 1).

Based on the simple Chi square test, female house sparrow visits were significantly more frequent at home prepared food and dog food than male visits, but water intake by female and male house sparrows did not differ significantly. However, the GLMM showed that only the type of food (Wald χ^2^ 11.47, df = 2, *p* = 0.003) significantly influenced the number of house sparrow visits (Table 3). Thus, based on the more conservative model which includes other variables and interactions, as well as the random effect, we did not find any significant difference between male and female visits. However, this model is based on the observed sex ratio of visits during our study and does not assume an equal sex ratio in the house sparrow population.

**Figure 2:**
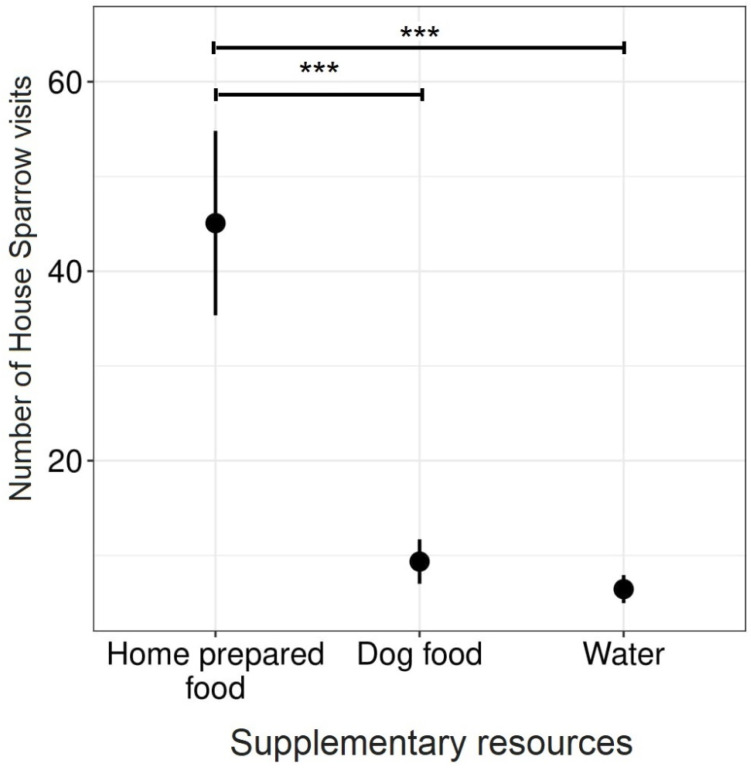
The mean ± standard error (SE) number of house sparrow visits per location to supplementary resources: home-prepared food, purchased dog food and water. *** indicates a significant difference at *p* < 0.001.

The number of visits was significantly higher when home prepared food was provided (mean ± SE 45.07 ± 9.73) than purchased dog food (9.35 ± 2.34) or water (6.44 ± 1.48) (Figure 2).

## 4. Discussion

Our results clearly show that the food provided for outdoor dogs is commonly used as a food resource by wild animals. We recorded feeding visits by wild animals of food or water intended for dogs in more than 70% of our study locations where dogs were fed outdoors. In combination with the large number of wild animal visits (see Table 1) this shows the importance of this resource for some species of wild animals. Species taking food and, less often, water are typical bird and mammal species living in Polish villages [5,29]. Most recorded visits were by the house sparrow. This synanthropic species is declining in Europe [18,19,20] and is unable to survive in the European environment unless in coexistence with human settlements [19,21]. This strong dependence of house sparrow populations is mostly based on artificial food resources generated by humans. However, current changes in housing of farm animals and in land use drastically limit the food resources available to house sparrows [30,31]. The traditional outdoor keeping and feeding of dogs is still popular in central and eastern Europe. They now provide one of the few remaining and predictable sources of human generated waste food in human settlements in the rural environment. This is different from the situation with most bird feeders, where food is often available less regularly, and mainly during harsh winters [10]. If this way of feeding dogs in Central Europe changes, this food source for the house sparrow will disappear and local extinctions of house sparrows should be expected. In England, the disappearance of overwinter food sources has been shown to be the cause for local population extinctions in rural habitats [18]. 

The time of year in which we monitored the activity of wild animals visiting dog feeding places is critical, especially for females building up body resources for the forthcoming breeding season. During this period, female sparrows collect additional energy resources in preparation for egg laying [32,33]. This is confirmed by a different use of food and water and is underlined by the significant difference in the number of visits between male and female house sparrows, despite the expected sex ratio balance in the population [34]. The second bird species regularly using the dog food was the magpie *Pica pica*. It is well known that this corvid uses artificial food sources in human settlements [35]. The frequency of magpie visits indicate that dog food is an important and well-used resource for this species allowing it to survive the winter in good condition. 

In contrast to bird visits, which can be considered as neutral or even as positive by some dog owners, rodent visits would rather be considered as a negative phenomenon. Rodent access to dog food occurs relatively often, and these resources can increase the reproduction rates of the brown rat, considered a most onerous pest.

However, each contact of wild animals with pets may contribute to the transfer of pathogens, especially if the faeces and secretions of wild animals reach the dogs’ food. Reported cases of pathogen transfer concern both wild birds to pets [36,37] and possible transfers from wild mammals to pets [38]. Wild animals visiting dog food dishes increases the probability of disease spreading, since it is the number of contacts of different individuals of wild animals that increase disease probability. House sparrows can transmit salmonellae [39], and the large numbers of recorded visits of this species during the current research suggests the possibility of relatively frequent transmission. Comparison of these findings with additional studies, focused on the *Campylobacter* species [40,41,42] suggest a more complicated situation. Firstly the dog dishes can stimulate the spread of pathogens between birds similar to bird feeders [42], secondly the dogs can be the source and reservoir from which wild animals become infected, especially since the reported prevalence of the *Campylobacter* species in dog populations is high [40,41].

## 5. Conclusions

The large number of wild animal visits shows the importance of dog food for some species of wild animal, especially house sparrows. The significant difference in the number of visits between male and female house sparrows suggests a critical role for females building up body resources for the forthcoming breeding season i.e., collecting additional energy resources in preparation for egg laying. For the house sparrow, which has declined throughout Europe, access to dog food is probably critical for the survival of local populations. 

Wild animals strongly preferred home-produced food rather than commercial dog food. This is interesting, because the production and use of commercial dog food, especially in developed countries, is increasing greatly [12,15]. Changes in the pattern of dog feeding are probably one further example of how human-generated food waste has affected bird numbers and behaviour [15], although it is probably only important at a local scale. On the other hand, taking account of global changes to the structure of villages, for example rather less associated with traditional farming but rather more as a dormitory settlement for city workers [3,5,14,43], our results may suggest a more general, even global, picture. 

As far as we are aware, this is the first study directly focused on this aspect, and before calculating the real environmental costs linked to changes in food production for pets (see [12]) more research is necessary. The recorded visits also increase the possibilities of pathogen transfer between wild animals and dogs, although at this stage this is mostly a speculative discussion.

## Figures and Tables

**Table 1:** Numbers of visits and frequency of feeding visits by wild animals recorded at 21 of 29 locations during 24 h monitoring. Species are arranged by descending frequency.

Species	Number of Visits			Frequency [%] of Feeding Visits
	Total	without Feeding	with Feeding	
House Sparrow *Passer domesticus*	1584	51	1533	87.4
Magpie *Pica pica*	122	8	114	6.5
Rodents	66	2	64	3.6
Tree Sparrow *Passer montanus*	28	1	27	1.5
Collared Dove *Streptopelia decaocto*	5	0	5	0.3
Great Tit *Parus major*	5	0	5	0.3
Red Fox *Vulpes vulpes*	3	0	3	0.2
Feral Pigeon *Columba livia f. urbana*	2	0	2	0.1
Jay *Garrulus glandarius*	2	1	1	0.1
Beech Marten *Martes foina*	1	0	1	0.1
	1818	63	1755	100.0

**Table 2:** The number of female and male house sparrow visits between the three food categories.

Category	Female	Male	χ^2^	*p*
Home prepared food	681	536	17.28	<0.001
Dog food	134	66	23.12	<0.001
Water	57	59	0.034	0.853

**Table 3:** Results of the generalized linear mixed model with a negative binomial distribution.

Variables	Wald χ^2^	df	*p*
Food type	11.47	2	0.003
Sex	0.08	1	0.781
Dog reaction	2.38	1	0.123
Food type × Sex	0.91	2	0.634
Food type × Dog reaction	3.59	2	0.166
Sex × Dog reaction	0.06	1	0.812

## Data Availability

The data presented in this study are available on request from the corresponding author. The data are not publicly available due to privacy reasons.
